# Employing zero-inflated beta distribution in an exposure-response analysis of TYK2/JAK1 inhibitor brepocitinib in patients with plaque psoriasis

**DOI:** 10.1007/s10928-024-09901-2

**Published:** 2024-03-03

**Authors:** Nikolaos Tsamandouras, Ruolun Qiu, Jim H. Hughes, Kevin Sweeney, John P. Prybylski, Christopher Banfield, Timothy Nicholas

**Affiliations:** 1 Clinical Pharmacology, Early Clinical Development, Worldwide Research, Development and Medical, Pfizer, Cambridge, MA USA; 2 Clinical Pharmacology, Global Product Development, Pfizer, Groton, CT USA

**Keywords:** Exposure-response modeling, Brepocitinib, Psoriasis, PASI, Bounded outcome scores, beta distribution

## Abstract

Brepocitinib is an oral selective dual TYK2/JAK1 inhibitor and based on its cytokine inhibition profile is expected to provide therapeutic benefit in the treatment of plaque psoriasis. Efficacy data from a completed Phase 2a study in patients with moderate-to-severe plaque psoriasis were utilized to develop a population exposure-response model that can be employed to inform dose selection decisions for further clinical development. A modeling approach that employs the zero-inflated beta distribution was used to account for the bounded nature and distributional characteristics of the Psoriasis Area and Severity Index (PASI) score data. The developed exposure-response model provided an adequate description of the observed PASI scores across all the treatment arms tested and across both the induction and maintenance dosing periods of the study. In addition, the developed model exhibited a good predictive capacity with regard to the derived responder metrics (e.g., 75%/90%/100% improvement in PASI score [PASI75/90/100]). Clinical trial simulations indicated that the induction/maintenance dosing paradigm explored in this study does not offer any advantages from an efficacy perspective and that doses of 10, 30, and 60 mg once-daily may be suitable candidates for clinical evaluation in subsequent Phase 2b studies.

**Supplementary Information:**

The online version contains supplementary material available at 10.1007/s10928-024-09901-2.

## Introduction

Psoriasis is estimated to affect more than 8 million people in the United States, and approximately 125 million people worldwide (2–3% of the global population) [1]. Although psoriasis primarily affects the skin and is not a life-threatening disease, it can profoundly impact the quality of life resulting in impairment analogous to other major diseases such as type 2 diabetes, myocardial infarction, and arthritis [2]. The most common variant of psoriasis, plaque psoriasis, is a chronic inflammatory disease characterized by red, scaly, raised plaques. Increased levels of several pro-inflammatory cytokines, including tumor necrosis factor-α (TNF-a), interleukin (IL)-6, IL-9, IL-12, IL-17, IL-22, and IL-23 and interferon gamma (IFN-γ) have been implicated in the pathophysiology of chronic plaque psoriasis [3, 4, 5].

Brepocitinib (PF-06700841) is an oral selective dual TYK2/JAK1 inhibitor targeting signaling of multiple cytokines (IFN, IL-6, IL-12, IL-21, IL-22, and IL-23) and based on its cytokine inhibition profile is expected to provide therapeutic benefit in the treatment of plaque psoriasis [6, 7]. In the first-in-human clinical trial, brepocitinib was well tolerated by healthy participants and patients with plaque psoriasis. Additionally, psoriasis patients receiving active treatment in this study (30 or 100 mg once daily for 28 days) had clinically meaningful decreases in disease activity [8]. Based on these data, a Phase 2a multicenter study was subsequently designed to assess efficacy, safety, and pharmacokinetics (PK) of oral brepocitinib in patients with moderate to severe plaque psoriasis, and test various doses and dosing regimens for induction and maintenance of brepocitinib (ClinicalTrials.gov Identifier: NCT02969018). This study has been now completed, and overall results showed a promising efficacy and a favorable safety profile in patients with moderate to severe plaque psoriasis [9].

One of the most commonly used efficacy assessments in psoriasis clinical trials is the Psoriasis Area and Severity Index (PASI). PASI score is a clinical tool that reflects both the extent (area) and severity of the disease and it can range from 0 (no evidence of the disease) to 72 (worst possible outcome) in 0.1 increments [10]. In addition, the PASI-derived responder metrics (e.g., PASI75 and PASI90 referring respectively to the proportion of patients achieving at least 75% or at least 90% improvement from baseline PASI scores) are commonly used primary endpoints. Due to their dichotomous nature and the fact that they omit the granularity of the raw PASI scores, the derived responder metrics may not be optimal for exposure-response assessment especially in small sample size early clinical development trials. Therefore, exposure-response analysis in the domain of the raw PASI scores is often desirable, especially if this can be also accompanied with satisfactory predictions of the derived responder metrics. However, modeling the PASI scores directly presents particular challenges as they are bounded between 0 and 72 and their distribution is often skewed. In addition, the obtained PASI datasets may often contain observations at the lower bound (0), particularly in the presence of a strong drug effect.

The primary aim of this work is to utilize the data derived from the completed Phase 2a study of brepocitinib in psoriasis patients to develop a population exposure-response model that can be employed to inform dose selection decisions for further clinical development. In parallel, this work aims to assess the utility of an approach that employs the zero-inflated beta distribution to overcome the challenges involved in PASI score modeling.

## Methods

### Study design

**Fig. 1:**
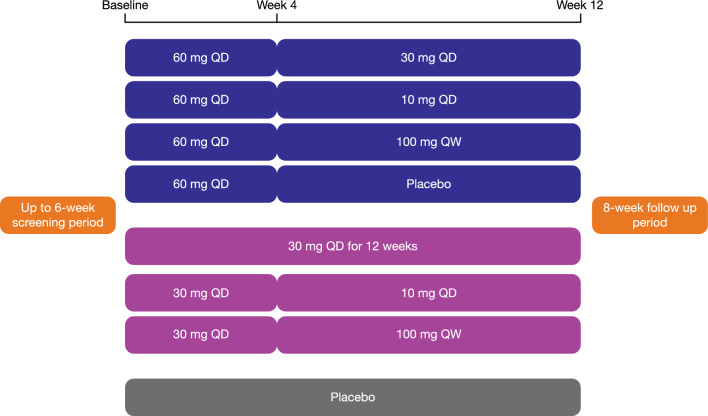
Study design schema. Schematic illustrates the eight different treatment arms of the study and the respective dosing regimens across induction (up to week 4) and maintenance (week 5 through 12) periods. *QD* once daily, *QW* once weekly

Model development was performed using data from a Phase 2a, randomized, double-blind, placebo-controlled, parallel group, multicenter study in subjects with moderate-to-severe plaque psoriasis (ClinicalTrials.gov Identifier: NCT02969018). A schematic of study design is shown in Fig. 1. The first part of the study, following a screening period (up to 6 weeks), was a 4-week induction period with double-blind daily treatment. At the end of week 4, all subjects switched to their predefined double-blind maintenance treatment regimen for week 5 through week 12.

Approximately 200 subjects were planned to be randomized into the study, to allow for approximately 160 evaluable subjects (20 completers per arm). The randomization ratio was 7:1, active: placebo. During the first 4 weeks of the treatment period, subjects received orally either 30 mg once-daily (QD), or 60 mg QD of brepocitinib, or matching placebo. During the 8-week maintenance portion of the treatment period (weeks 5 through 12), subjects received orally either 10 mg QD, or 30 mg QD, or a 100 mg once weekly (QW) regimen of brepocitinib, or matching placebo. Maintenance dose level and regimen were assigned at the initial time of randomization into the study. All subjects, regardless of assigned regimen (i.e., QD or QW) received blinded QD tablets throughout the study treatment period to maintain the study blind.

The duration of study subject participation was approximately 26 weeks, including screening (up to 6 weeks), 12-week treatment period, and 8-week follow up period. For further details on the study design, the reader is referred to the associated publication [9].

Female and male patients between 18 and 75 years of age with a diagnosis of plaque psoriasis for at least 6 months before the start of the study, PASI score of ≥ 12, physician’s global assessment (PGA) score of 3 or 4, and psoriasis covering ≥ 10% of total body surface area were eligible to participate in the study. Key exclusion criteria were non-plaque psoriasis, other skin conditions that would affect the assessment of psoriasis, drug-induced psoriasis, use of corticosteroids, and psychiatric conditions including suicidal ideation or behavior.

The study was conducted in compliance with the Declaration of Helsinki and Good Clinical Practice Guidelines established by the International Council on Harmonisation. The final protocol, amendments, and informed consent documentation were reviewed and approved by the institutional review boards and independent ethics committees of the investigational centers.

### Study assessments

PASI scoring, which quantifies body surface area and lesion severity into a single score, was used to assess brepocitinib efficacy in this study. PASI score is calculated by combining the percentage of body areas (head and neck, upper limbs, trunk, and lower limbs) covered, with the severity of erythema, thickness/induration, and desquamation/scaling, for a score between 0 and 72 [10]. PASI scores were assessed: at screening; on week 0 (baseline); on weeks 1, 2, 4, 6, 8, 10, and 12; and during follow-up (weeks 14 and 16). Only data collected during the 12-week active treatment period of the study were included in the analysis. Any data collected during screening and the follow-up period were not included in the analysis.

Blood samples for PK analysis of brepocitinib were collected pre-dose on week 0 (baseline) and week 1; pre-dose and 30 min post-dose on weeks 2, 6, 8, and 10; and pre-dose, 30 min, 1, 2, and 4 h post-dose on weeks 4 and 12. The samples were analyzed using high-performance liquid chromatography-tandem mass spectrometry. The lower limit of quantification for brepocitinib was 0.2 ng/mL.

For additional details and study assessments not related to the current analysis, the reader is referred to the associated publication [9].

### Population PK model and derivation of C_ave_ for exposure-response modeling

A population pharmacokinetic (PK) model has been previously developed for brepocitinib using data from five clinical trials, consisting of three Phase 1 and two Phase 2 studies (including the Phase 2a study in psoriasis patients described in this manuscript) [11]. Briefly, brepocitinib PK were described with a one-compartment model with first-order oral absorption and an absorption lag for the tablet formulation (apparent clearance (CL/F) of 18.7 L/h, apparent volume of distribution (V/F) of 136 L, first-order absorption rate constant (k_a_) of 3.46 h^−1^ and a lag time (A_lag_) of 0.24 h). The effect of body weight on CL/F and V/F was included with an allometric relationship (referenced to 70 kg) and the associated coefficients were fixed to 0.75 and 1, respectively. Random inter-individual variability was accounted in the CL/F and V/F parameters using a full variance-covariance matrix (coefficient of variation (CV) of 78% and 60.5% for CL/F and V/F, respectively, and a correlation coefficient of 0.76).

The empirical Bayes estimates (EBEs) of CL/F for all study participants (η-shrinkage < 0.1%) were extracted from the population PK model output and were used to calculate the average concentration (${C}_{ave}$) of each individual across the induction and maintenance periods of the study, using Eq. 1, where $\tau$ refers to the dosing interval (24 h for QD dosing and 168 h for QW dosing) and $Dose$ refers to the administered brepocitinib dose either during the induction or the maintenance period of the study.

1 $${C}_{ave}=\frac{Dose}{\left(CL/F\right)\cdot \tau } $$

The derived ${C}_{ave}$ for each study participant was the exposure-relevant metric that was used in the exposure-response analysis (see Population exposure-response model section, Eq. 12). Brepocitinib PK is assumed to be at steady state during all efficacy assessments performed over the active treatment period of the study (since brepocitinib has a short terminal half-life, ranging from 3.8 to 7.5 h, and efficacy assessments were performed at least 1 week since first dose either during the induction or maintenance period).

### Population exposure-response model

An approach that employs a zero-inflated beta distribution [12, 13] was used to allow modeling of PASI scores without disregarding their bounded nature (range from 0 to 72). To enable this, observed PASI scores were first transformed to the 0 to 1 interval using Eq. 2.

2 $$y=\frac{PASI}{72}$$

**Fig. 2:**
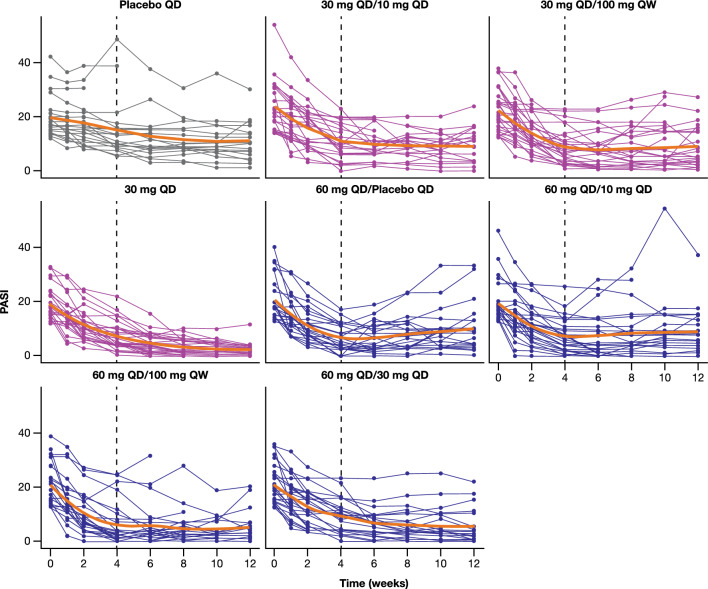
Longitudinal individual PASI profiles stratified by treatment arm. The vertical dashed black line at 4 weeks highlights the transition from the induction to the maintenance treatment period. The solid orange line represents *loess* smoothing of the data. When a different dose is administered between induction (up to week 4) and maintenance (weeks 5 through 12) periods the respective regimens are reported separated by “/”. *PASI* Psoriasis Area and Severity Index, *QD* once daily, *QW* once weekly

Note that the analyzed dataset although it contains observations on the lower bound (PASI = 0), it does not contain any observations on the upper bound (PASI = 72), see Fig. 2, thus $y\in \left[0,\right.\left.1\right)$. In the case that the dataset had contained data on both boundaries, the use of a zero- and one-inflated beta distribution [12, 13] would have been appropriate.

The transformed PASI scores ($y$) were then assumed to follow a zero-inflated beta distribution with a corresponding probability density function that is given by the mixture in Eq. 3,

3 $$p\left(y;\, {p}_{0},\,\alpha ,\,\beta \right)=\left\{\begin{array}{c}{p}_{0} ,i\!f \,\,y=0\\ \left(1-{p}_{0}\right)\cdot f\left(y;\alpha ,\beta \right) , i\!f \,\,0<y<1\end{array}\right.$$

where ${p}_{0}$ is the probability of a 0 observation (i.e., PASI = 0) and $f\left(y;\alpha ,\beta \right)$ is the density function of the beta distribution defined in Eq. 4,

4 $$f\left(y;\alpha ,\beta \right)=\frac{\varGamma \left(\alpha +\beta \right)}{\varGamma \left(\alpha \right)\cdot \varGamma \left(\beta \right)}\cdot {y}^{\alpha -1}\cdot {\left(1-y\right)}^{\beta -1} $$

where $\Gamma$ (∎) is the *gamma* function and $\alpha$ and $\beta$ are shape parameters of the beta distribution (with $\alpha$ > 0 and $\beta$ > 0). The Nemes approximation to the *gamma* function was used [14, 15, 16], as defined in Eq. 5, where ${\rm X}$ represents $\alpha$, $\beta$, or $\alpha +\beta$ in the density function above (note that the *GAMLN* function can alternatively be used in NONMEM^®^ Version 7.3 [17] onwards).

5 $$\varGamma \left({\rm X}\right) \sim {\left(\frac{{\rm X}}{e}\right)}^{X}\cdot \sqrt{\frac{2\pi }{X}}\cdot {\left(1+\frac{1}{15{X}^{2}}\right)}^{\frac{5}{4}X}$$

The shape parameters $\alpha$ and $\beta$ were parameterized (see Eqs. 6 and 7) with respect to the expected value of the beta distribution $\mu$ and the precision parameter $\phi$.

6 $$\alpha =\mu \cdot \phi $$

7 $$\beta =\left(1-\mu \right)\cdot \phi$$

Under this parameterization, the variance (${\sigma }^{2}$) of the beta distribution is defined in Eq. 8. Thus, the parameter $\phi$ plays the role of a precision parameter in the sense that for a given value of $\mu$, variance decreases as the value of $\phi$ increases.

8 $${\sigma }^{2}=\frac{\mu \cdot \left(1-\mu \right)}{\phi +1}$$

The precision parameter $\phi$ was estimated as a fixed effect parameter, while $\mu$ was parameterized (see Eq. 9) with respect to baseline PASI score ($BSL$), the placebo effect (${f}_{p}\left(t\right)$) and the drug effect (${f}_{d}\left(t\right)$).

9 $$\mu =\frac{1}{72}\cdot \left(BSL-{f}_{p}\left(t\right)-{f}_{d}\left(t\right)\right) $$

The placebo effect (${f}_{p}\left(t\right)$) was modeled as illustrated in Eq. 10 with an empirical relationship, where ${P}_{max}$ represents the maximum placebo effect (expressed as fraction of baseline), and ${k}_{p}$ represents the rate of onset of the placebo effect.

10 $${f}_{p}\left(t\right)=BSL\cdot {P}_{max}\cdot \left(1-{e}^{-{k}_{p}\cdot t}\right) $$

The drug effect (${f}_{d}\left(t\right)$) was modeled as illustrated in Eq. 11 through a latent variable $R$ on which the drug elicits its effect via a Type I indirect response model (Eq. 12), similarly to [18].

11 $${f}_{d}\left(t\right)=BSL\cdot \left(1-{P}_{max}\right)\cdot \left(1-R\left(t\right)\right) $$

12 $$\frac{dR\left(t\right)}{dt}={k}_{in}\cdot \left(1-\frac{{C}_{ave}}{{IC}_{50}+{C}_{ave}}\right)-{k}_{out}\cdot R\left(t\right)$$

It was further assumed that at baseline the latent variable $R$ takes the value of 1 (i.e., $R\left(0\right)=1$) and consequently ${k}_{in}={k}_{out}$ (i.e., equal rates of onset and offset of the drug effect).

The model parameterization described in Eq. 9, 10, 11, 12 assures that $\mu$, as the expected value of the beta distribution, is constrained within the $\left(\text{0,1}\right)$ interval. The value of $\mu$ can be interpreted as the individual PASI score prediction after transformation to the $\left(\text{0,1}\right)$ domain (Eq. 9).

The probability of a 0 observation (${p}_{0}$), see Eq. 3, was modeled through Eq. 13 as a function of $\mu$ and two additional parameters ${\zeta }_{1}$ and ${\zeta }_{2}$ (with ${\zeta }_{2}$ > 0). The rationale for this parameterization is to enforce through a flexible function that the smaller the value of $\mu$ for a given subject, the higher the probability of an actual PASI=0 observation.

13 $${p}_{0}=\frac{{e}^{\left({\zeta }_{1}-{\zeta }_{2}\cdot \mu \right)}}{1+{e}^{\left({\zeta }_{1}-{\zeta }_{2}\cdot \mu \right)}} $$

Incorporation of random effects on the parameters of the exposure-response model was assessed using different parameterizations and covariance structures in order to take into account inter-individual variability in the observed response. Since the ${P}_{max}$ and $BSL$ parameters need to be constrained at the individual level within a bounded region, inter-individual variability on these parameters was assessed using a generalization of the logit-normal distribution (see Eq. 14) [19],

14 $${P}_{i}={B}_{l}+\left({B}_{u}-{B}_{l}\right)\cdot \frac{{e}^{log\left(\frac{{\theta }_{p}-{B}_{l}}{{B}_{u}-{\theta }_{p}}\right)+{\eta }_{i}}}{1+{e}^{log\left(\frac{{\theta }_{p}-{B}_{l}}{{B}_{u}-{\theta }_{p}}\right)+{\eta }_{i}}} $$

where ${P}_{i}$ is the individual value of parameter $P$, ${\theta }_{p}$ is the typical population value of parameter $P$, ${\eta }_{i}$ is the random effect term with respect to the inter-individual variability in parameter $P$ assuming to follow a normal distribution with mean of 0 and variance ${\omega }^{2}$, and ${B}_{l}$ and ${B}_{u}$ are the lower and upper bounds respectively of parameter $P$. ${B}_{l}$ and ${B}_{u}$ were set to 0 and 1 respectively for ${P}_{max}$ (since it represents a fraction) and to 12 and 72 for $BSL$ (since baseline PASI score cannot be less than 12 per study’s inclusion criteria).

No covariates were tested in the current exposure-response analysis.

### Assessment of model performance

In addition to evaluating the relative standard errors derived from the NONMEM^®^ covariance step, the parameter uncertainty of the final model was also assessed using sampling importance resampling (SIR) [20, 21]. SIR was performed by subjecting the final model covariance step output to five iterations of sampling, resampling, and multivariate Box-Cox transformation. Samples for the sampling and resampling steps used 1000, 1000, 1500, 2000, 2000 and 200, 250, 500, 1000, 1000 samples, respectively. The median and 95% confidence intervals (CIs) for each parameter were calculated from the SIR resamples of the final iteration.

The model’s performance to adequately describe the observed PASI scores was assessed via a visual predictive check (VPC) based on 1000 simulations using the design of the index dataset. Additionally, although the model was developed using the raw PASI scores, it is of particular importance to be also able to describe derived responder metrics, as the latter are important endpoints for future studies. Therefore, the model performance was also evaluated with respect to its capacity to predict the following responder metrics: proportion of participants achieving at least 50%, 75%, 90%, and 100% improvement from baseline PASI (PASI50, PASI75, PASI90, and PASI100, respectively). For this evaluation these responder metrics were derived from the raw simulated PASI scores in each of the 1000 simulated datasets, and associated 95% CIs were generated. The latter were then compared to the responder metrics that were observed in the current study.

### Clinical trial simulations for dose selection in future studies

The developed population exposure-response model was used to perform clinical trial simulations to aid dose selection for subsequent trials. Variability in brepocitinib exposure was accounted by sampling $CL/F$ from the associated population distribution determined in the population PK model after incorporating the effect of weight (weight of each simulated individual was randomly sampled with replacement from the current study dataset). The individual $CL/F$ values were subsequently used to calculate ${C}_{ave}$ for each simulated individual using Eq. 1. The output ${C}_{ave}$ values were then passed to the developed population exposure-response model to simulate PASI score response in clinical trials.

A set of 2000 clinical trials were simulated assuming that brepocitinib or placebo were administered QD for 16 weeks. Each participant was assumed to be assigned the same treatment throughout a 16-week period (no induction/maintenance periods). Brepocitinib dose levels between 5 and 60 mg were evaluated in 5 mg intervals. Each treatment arm (either brepocitinib or placebo) was assumed to have a sample size of 56 participants. Efficacy assessments (PASI scores) were assumed to be performed at baseline and every 2 weeks until week 16.

The PASI75, PASI90, and PASI100 responder metrics after 16 weeks of treatment are expected to be the endpoints of interest in subsequent studies. Thus, the output from the clinical trial simulations (raw PASI scores) was summarized to derive predictions for these responder metrics on week 16 together with associated 90% CIs across all the evaluated dose levels.

Using the exact framework described above, additional exploratory clinical trial simulations were performed using selected dose levels in order to compare the projected efficacy of flat dosing (same dose throughout the 16-week period) with that of an induction/maintenance dosing paradigm (4 weeks of induction dose followed by 12 weeks of maintenance dose).

### Modeling software

The population exposure-response model was developed using non-linear mixed effects methods and NONMEM^®^ Version 7.3 [17]. Population parameter estimation was performed with the Laplacian estimation method. The ADVAN13 subroutine with TOL = 7 was used for solving differential equations. NONMEM^®^ control stream is provided in Online Resource 1.

SIR was conducted using Perl speaks NONMEM^®^ (PsN) (version 5.2.6) [22, 21]. Data visualization, exploratory analyses, model diagnostics, post-processing of NONMEM^®^ output and all clinical trial simulations were generated using the R statistical and programming language [23] (version 3.6.1).

## Results

### Data description

Overall, 212 patients were randomized and received at least one dose of brepocitinib or placebo. Of the 212 participants, 148 (69.8%) were male and 64 (30.2%) female. 189 subjects (89.2%) were White, 11 (5.2%) were Black or African American, four (1.9%) were Asian and eight (3.8%) were identified as other race. The median age in the study participants was 48 years (ranged from 18 to 75 years), and the median weight was 91.6 kg (ranged from 45.1 to 204.3 kg).

The median baseline PASI score across all study participants was 18.2 (ranged from 12 to 54). An overview of the number of subjects and the mean baseline PASI score in each treatment arm is presented in Table 1. The observed longitudinal PASI score profiles of all study participants, stratified across the different treatment arms, are illustrated in Fig. 2.

**Table 1:** Number of participants and mean baseline PASI in each treatment arm

Treatment	N randomized	Mean baseline PASI (SD)	N completed
Placebo QD	23	19.6 (7.6)	17
30 mg QD/10 mg QD	25	23.8 (9.1)	19
30 mg QD/100 mg QW	30	21.7 (7.5)	24
30 mg QD	29	19.1 (5.9)	27
60 mg QD/Placebo QD	25	20.6 (8.2)	21
60 mg QD/10 mg QD	29	19.3 (7.5)	21
60 mg QD/100 mg QW	26	20.7 (7.9)	20
60 mg QD/30 mg QD	25	21.5 (7.5)	21

*N randomized* number of participants randomized in each treatment arm, *N completed* number of participants who completed the 12-week treatment period in each treatment arm, *PASI* Psoriasis Area and Severity Index, *QD* once daily, *QW* once weekly, *SD* standard deviation

When a different dose is administered between induction (up to week 4) and maintenance (weeks 5 through 12) periods the respective regimens are reported separated by “/”

The median baseline PASI score across all study participants was 18.2 (ranged from 12 to 54)

A total of 170 of the 212 randomized participants completed the 12-week treatment period of the study (Table 1). Patient flow and a disposition diagram, including reasons for discontinuation, are reported in the primary manuscript [9]. The investigation of the longitudinal individual PASI profiles indicated no substantial differences overall in the efficacy trajectories between participants who dropped out before the end of the 12-week treatment period and those who completed the 12-week treatment period (Online Resource 2). Therefore, dropout was assumed to have no impact on model development and estimation-based inferences. For simulation-based diagnostics of model performance (e.g., VPC), the exact same structure of the index (true) dataset was used, and, as such, the simulated datasets had identical dropout with the index (true) dataset.

### Population exposure-response model results

The parameter estimates of the developed population exposure-response model are reported in Table 2. All model parameters were estimated with acceptable precision. The estimates for the rate of onset of the placebo and the drug effect are aligning with the observation that several weeks on-treatment are needed for the observed response to start plateauing. Inter-individual variability was incorporated only with regard to $BSL$ (baseline PASI score), as models with additional variability components on other parameters faced convergence issues.

**Table 2:** Parameter estimates of the population exposure-response model

Parameter	Estimate	RSE (%)	SIR median (95% CI)
$BSL$	18.00	2.69	17.98 (16.89–19.08)
${IC}_{50} \left(ng/mL\right)$	53.24	21.43	53.21 (43.72–65.69)
${k}_{out} \left({Day}^{-1}\right)$	0.067	13.42	0.067 (0.056–0.081)
${P}_{max}$	0.489	8.41	0.489 (0.447–0.528)
${k}_{p} \left({Day}^{-1}\right)$	0.047	6.48	0.047 (0.042–0.053)
$\phi$	31.25	8.74	31.10 (28.87–34.00)
${\zeta }_{1}$	2.228	39.95	2.241 (1.357–3.259)
${\zeta }_{2}$	62.67	20.24	62.83 (49.40–79.77)
${\eta }_{BSL} \left({\omega }^{2}\right)$	1.655	12.07	1.682 (1.297–2.169)

*CI* confidence interval, *RSE* relative standard error, SIR: sampling importance resampling

RSE (%) is the relative standard error of the parameter estimate calculated as: $\left(standard \,\,error/estimate\right) \times 100$

Standard errors were derived from NONMEM^®^ covariance step

SIR median and 95% CIs calculated from the 50th, 2.5th, and 97.5th percentiles for the distribution of 1000 resamples from the 5th SIR iteration of the final model

As described in Methods, it is assumed that *k*_*in*_ = *k*_*out*_

**Fig. 3:**
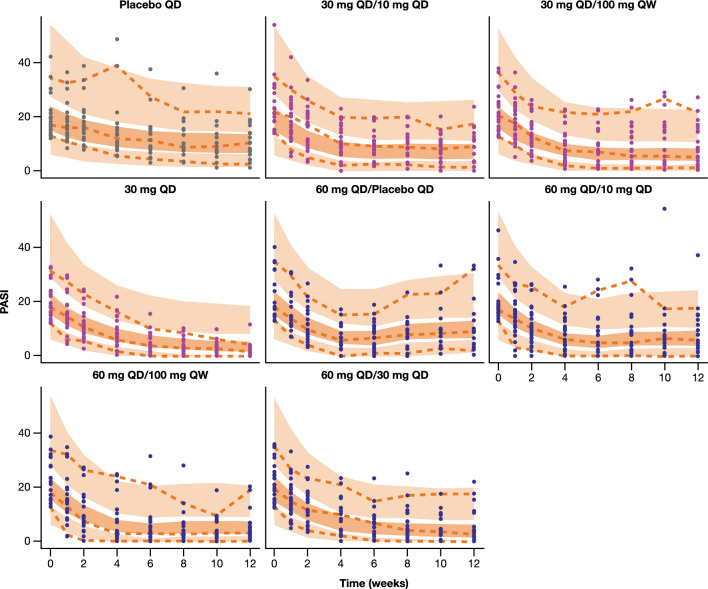
VPC stratified by treatment arm. Closed circles represent the observed individual PASI data. Orange dashed lines represent the 5th, 50th, and 95th percentile of the observed data. Dark orange and light orange shaded areas represent 95% CIs around the model-derived median and model-derived 5th/95th percentiles, respectively. *PASI* Psoriasis Area and Severity Index, *QD* once daily, *QW* once weekly, *VPC* visual predictive check

Model diagnostics supported the final model selected. Assessment of the model’s performance via VPC indicated that the selected model adequately captured the observed PASI data and the associated variability across all treatment arms and across both induction and maintenance periods (Fig. 3). Alternative VPC plot illustrating 10th/90th percentiles instead of 5th/95th percentiles (given the relatively small sample size per treatment arm) is provided in Online Resource 3. Characteristically, the model was able to adequately capture PASI trajectories both in the arms that had the same treatment across the 12-week period (e.g., Placebo QD, 30 mg QD) but also in the arms that switched treatments between induction and maintenance periods. In some of the latter arms (e.g., 60 mg QD/Placebo QD or 60 mg QD/10 mg QD) a trend of rebound of the disease or flattening of the drug response was generally observed and this trend was successfully captured by the model.

Individual model fits (observed and predicted PASI scores over time) from 36 randomly sampled study participants are presented in Online Resource 4.

**Fig. 4:**
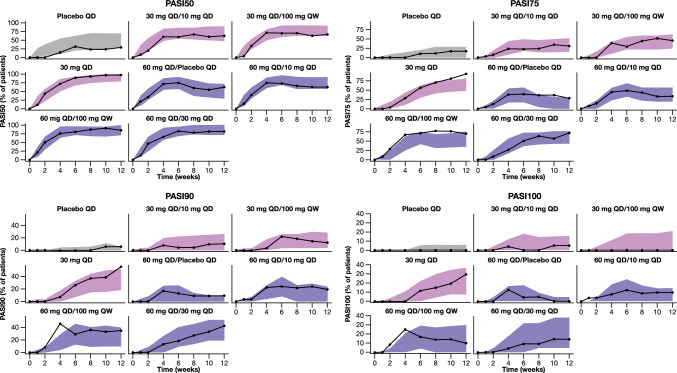
Observed and predicted responder metrics stratified by treatment arm. Solid black line with closed black circles represents the observed PASI50 (top left), PASI75 (top right), PASI90 (bottom left), and PASI100 (bottom right) responder metrics in the study. Shaded areas represent the associated 95% CIs around the model prediction. PASI 50/75/90/100: 50%/75%/90%/100% improvement in Psoriasis Area and Severity Index score, *QD* once daily, *QW* once weekly

In addition, the model was able to adequately capture not only the observed raw PASI scores but also the derived responder metrics (Fig. 4). When taking into account the totality of the data across all treatment arms, the observed trajectories were in good agreement with the associated model-derived 95% CIs across all the evaluated responder metrics.

### Clinical trial simulation results for dose selection in future studies

**Fig. 5:**
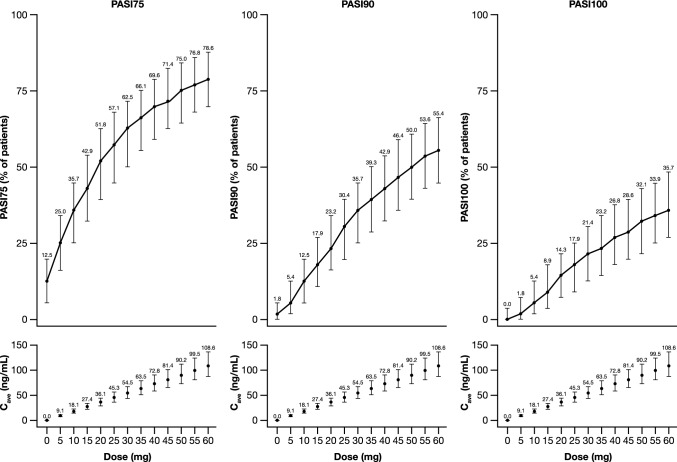
PASI75, PASI90, and PASI100 responder metrics on week 16 across different dose levels as predicted from clinical trial simulations. Top panels: PASI75 (left), PASI90 (center), and PASI100 (right) on week 16 for different dose levels of brepocitinib (QD), as predicted from clinical trial simulations (assuming same dose throughout the 16-week period). Bottom panels: Population median C_ave_ across different dose levels of brepocitinib (QD). In the top and bottom panels, error bars represent simulation-based 90% CIs around the prediction, and points refer to the median prediction. The median prediction is also numerically reported on top of each error bar. Top and bottom panels have a shared x-axis and Dose of 0 refers to placebo. C_ave_, average concentration; PASI 75/90/100: 75%/90%/100% improvement in Psoriasis Area and Severity Index score, *QD* once daily

The predictions for the PASI75, PASI90, and PASI100 responder metrics on week 16 across different dose levels (assuming same dose throughout the study and no induction/maintenance periods), as derived from the clinical trial simulations, are presented in Fig. 5. These results indicate that a dose of 10 mg QD for 16 weeks is the lowest practical dose with an effect on PASI75 and PASI90 that could be distinguished from placebo (non-overlapping CIs, see Fig. 5). A dose of 30 mg QD appears to provide a robust response at 16 weeks as 62.5%, 35.7%, and 21.4% of patients will have at least 75% (PASI75), 90% (PASI90), and 100% (PASI100) improvement, respectively, from their baseline PASI scores. Finally, a dose of 60 mg QD administered throughout for 16 weeks appears to provide meaningful numerical improvement in response compared to the 30 mg QD dose, as it will produce a PASI75, PASI90, and PASI100 of 78.6%, 55.4%, and 35.7%, respectively. The three dose levels discussed above (10, 30, and 60 mg QD) produce a median ${C}_{ave}$ of 18.1, 54.5, and 108.6 ng/mL which correspond to 0.34 times, 1.02 times, and 2.04 times, respectively the IC_50_ determined in the current analysis (53.24 ng/mL).

**Fig. 6:**
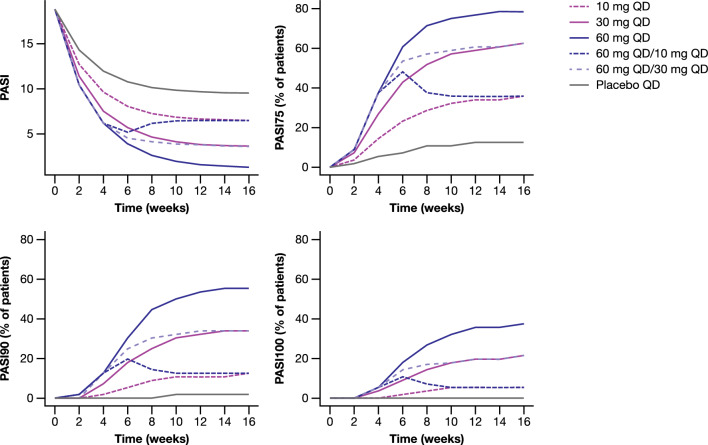
Longitudinal profiles of PASI scores and PASI75, PASI90, and PASI100 responder metrics as predicted from clinical trial simulations to compare flat dosing (same dose throughout the 16-week period) with an induction/maintenance dosing paradigm. For the induction/maintenance regimens, 4 weeks of induction (60 mg QD) followed by 12 weeks of maintenance (30 or 10 mg QD) has been assumed. Predictions are summarized as the median over 2000 simulations. PASI: Psoriasis Area and Severity Index, PASI 75/90/100: 75%/90%/100% improvement in PASI score, *QD* once daily

Additional exploratory simulations were performed to compare the projected efficacy of flat dosing (10, 30 or 60 mg QD throughout the 16-week period) with that of an induction/maintenance dosing paradigm (4 weeks of induction with 60 mg QD followed by 12 weeks of maintenance dose with 10 or 30 mg QD). The output from these simulations regarding the longitudinal profiles of PASI scores and responder metrics of interest (PASI75, PASI90, and PASI100) is presented in Fig. 6. It is apparent that an induction/maintenance paradigm offers no benefits from an efficacy perspective as both the “60 mg QD/30 mg QD” and “60 mg QD/10 mg QD” regimens provide an efficacy trajectory that by week 16 is converging to that achieved with flat dosing of 30 and 10 mg QD, respectively.

## Discussion

In this Phase 2a trial, patients with moderate-to-severe plaque psoriasis received 30 mg QD or 60 mg QD of brepocitinib or placebo for a 4-week induction period, followed by 10 mg QD or 30 mg QD or 100 mg QW of brepocitinib or placebo for an 8-week maintenance period. Based on the primary endpoint analysis of this trial, statistically significant differences in change from baseline PASI at week 12 were detected in all seven studied regimens with the exception of the “60 mg QD/10 mg QD” and “60 mg QD/Placebo QD” treatments, compared to placebo group [9]. The value of the exposure-response analysis presented here is that it allows not only to understand the longitudinal trajectory of the observed PASI scores and the association with drug exposure but also to bring together in a quantitative context data observed across different treatment regimens and induction/maintenance periods. The developed model provided an adequate description of the observed PASI data across all the different dose regimens tested and across both induction and maintenance periods, while it also had overall a good predictive capacity with regard to the derived responder metrics (e.g., PASI75, PASI90). This enabled the use of model-based clinical trial simulations to predict responder metrics and guide dose selection for future trials (e.g., Phase 2b) that may evaluate a different treatment duration (e.g., 16 weeks) or different treatment regimens compared to what was tested in the current study. With integration of the observed longitudinal data, the model suggested both placebo and drug effect to be plateauing by 12 weeks, and given the accurate estimation of the associated model parameters, extrapolation beyond 12 weeks is justifiable.

The rationale of the current study for the 4-week induction period was to assess whether an induction and maintenance dosing regimen provided a better efficacy and safety profile than continuous treatment with the same dose and whether the reduction in the dose or dosing frequency could maintain the clinical response achieved during the induction period. Based on the efficacy results in this study and as further confirmed by model simulations it was apparent that due to the pharmacokinetic (short half-life) and pharmacodynamic (onset/offset of drug effect) characteristics of this compound, the induction/maintenance paradigm is not advantageous compared to traditional flat dosing regimens where the same dose is administered throughout the study. Such an induction/maintenance approach may have been advantageous for a compound with a long half-life, slower offset rate of the drug effect, or in the case that the induction regimen achieves a complete re-equilibration of the disease and the underlying inflammatory tone. A flat dose paradigm should be preferred in future studies with brepocitinib in the absence of any safety concerns that may require minimization of the timeframe that a participant is exposed to a given dose. Based on clinical trial simulations presented here, doses of 10, 30, and 60 mg QD throughout the 16-week treatment period are emerging as suitable candidates for clinical evaluation in subsequent Phase 2b dose-ranging studies. It should be noted that, in the current study, 30 mg QD was the maximum dose administered throughout the 12-week treatment period, and the 60 mg QD dose was administered in some treatment arms for the initial 4-week induction period only. Then, simulations with doses greater than 30 mg QD throughout the 16-week treatment period represent an extrapolation from the current study dataset, and, as such, should be interpreted with caution and require confirmation in future trials.

Direct modeling of PASI data has particular challenges due to them being bounded outcome scores defined over the closed 0 and 72 interval. As such, these data may be often skewed exhibiting non-standard distributions (e.g., L-shaped in the presence of a strong drug response), while their error distribution may need to be heteroscedastic (since the variance may be decreasing approaching 0 close the lower boundary) [15]. Therefore, traditional modeling approaches assuming normality neglect the bounded nature of the data, may be inadequate to capture their distributional characteristics and may predict scores outside their nominal range. Several approaches have been proposed in the literature to handle bounded outcome score data [24, 25, 26, 27, 28], and Hu has recently written an elegant review discussing, comparing and providing guidance for the application of such methods in pharmacometrics [29].

The beta distribution due to its unique characteristics and flexibility of its density function can be a valuable tool for the analysis of bounded outcome scores and a beta-regression approach has been previously applied particularly in the area of Alzheimer’s disease scores [15, 16, 30]. One of the limitations of this methodology is that when the analyzed dataset contains scores that are exactly at the boundary (e.g., 0 or 72 for PASI), data need to be transformed/rescaled using an arbitrary small correction factor δ to map the data inside the open 0 to 1 interval. It has been illustrated before [30] and it is also the authors’ experience that modeling results can be sensitive on the choice of δ, thus raising doubts on the interpretation of the model output and highlighting the need for additional sensitivity analyses. In addition, the derived model cannot output scores exactly at the boundary (e.g., PASI of 0) when used for clinical trial simulations. This is an important shortcoming especially for compounds exhibiting a robust drug response where projection of the proportion of patients achieving complete remission (e.g., PASI100) is of particular interest.

Use of an inflated beta distribution [12, 13] has the potential of alleviating these limitations of the traditional beta distribution as it employs a mixture that involves the beta density for the non-boundary data and a probability mass associated with data exactly at the boundaries. As such, there is no need for rescaling data at the boundaries during analysis, while also scores exactly at the boundaries can be predicted in clinical trial simulations. This approach was employed here to analyze the exposure-response relationship of PASI scores from a Phase 2a trial in psoriasis patients and subsequently perform simulations to inform dose selection decisions for further clinical development. The value of this methodology for such an application lies in the fact that it respects the bounded nature of the data and its suitability was illustrated with the adequate model description of the observed PASI scores. Additionally, it has been previously stressed [31, 29] that models developed on the domain of the observed PASI scores often have limited capacity to also accurately predict the associated responder metrics. In this work, the developed PASI score model provided a good overall description across these derived metrics (PASI50, PASI75, PASI90, and PASI100). This provides further evidence that the modeling approach used here that employs the zero-inflated beta distribution is suitable to capture the distributional characteristics of the observed PASI data not only away but also near or at the boundary and thus providing accurate translation to the derived responder metrics. This is of particular importance as it enabled the exposure-response analysis to be performed in the domain of the actual PASI scores which offer the desired granularity in drug response, while also being confident on projections associated with the responder metrics that may be the primary endpoints in Phase 2b/3 trials. Three distinct approaches from what was used here, the latent beta variable approach [32, 33], the combined uniform and binomial approach [32, 33], and the bounded integer model [34], have been recently proposed for PASI score modeling, also exhibiting promising results.

PASI scores range from 0 (no evidence of the disease) to 72 (worst possible outcome) in 0.1 increments [10], and thus, PASI data are technically discrete (containing both integer and non-integer values). The approach/model presented in this study, outputs a continuous prediction for PASI scores that can take any value between 0 and 72 (or PASI being exactly 0), rather than producing values exactly at 0.1 increments. However, given the large number of possible categories and high granularity of the PASI scale (nearly continuous), the presented approach is fit for purpose and does not carry any impact on clinical decision-making.

This work is not without limitations. (1) No covariates were tested in the current exposure-response analysis. Covariate effects will need to be explored in future trials where larger sample size and more heterogenous data will be available. (2) A sequential modeling approach was employed here where subject-level ${C}_{ave}$ derived from a population PK analysis was assumed to be the exposure-relevant metric that drives response. It is not known whether simultaneous modeling of the full PK/PD profiles will offer any substantial advantages. (3) The available data could not support estimation of inter-individual variability components (random effects) in most of the parameters of the exposure-response model and thus inter-individual variability was incorporated only with regard to $BSL$ (baseline PASI score). Thus, the observed population variability in response, although adequately captured, could not be precisely allocated to specific mechanisms/sources (e.g., onset of drug effect). (4) Uncertainty on the model parameter estimates was not taken into account in simulations. However model parameters were estimated with relatively high precision and thus the associated impact of parameter uncertainty was expected to be minimal and not meaningfully affecting any decision making for the clinical development of brepocitinib. (5) Only data collected during the 12-week active treatment period of the study were included in the exposure-response model to align with the primary endpoint analysis (change from baseline at Week 12). Inclusion of PASI data collected during the follow-up period (after the end of treatment) may provide additional information regarding the rate of offset of drug effect and will be considered in future brepocitinib exposure-response analyses. (6) Finally, the simulations performed in this work with the scope of dose selection for further clinical development focus solely on an efficacy perspective. The safety and tolerability data of brepocitinib [8, 9] are equally important and should be considered in conjunction with the projected efficacy outcomes.

## Conclusions

The developed population exposure-response model for brepocitinib provided an adequate description of the observed PASI score data, while it also had a good predictive capacity with regard to the derived responder metrics (e.g., PASI75, PASI90). Clinical trial simulations with the developed model indicate that brepocitinib doses of 10, 30, and 60 mg QD may be suitable for clinical evaluation in subsequent Phase 2b studies.

### Supplementary Information

Below is the link to the electronic supplementary material.

Supplementary material 1 (DOCX 1674 kb)
