# Supplementary material for: Employing zero-inflated beta distribution in an exposure-response analysis of TYK2/JAK1 inhibitor brepocitinib in patients with plaque psoriasis
Source: J Pharmacokinet Pharmacodyn. 2024 Mar 3;51(3):265–77. doi: 10.1007/s10928-024-09901-2 (PMC11136736; doi:10.1007/s10928-024-09901-2)
Supplement: Supplementary file 1 — Supplementary material 1 (DOCX 1674 kb) [file 10928_2024_9901_MOESM1_ESM.docx]

Employing zero-inflated beta distribution in an exposure-response analysis of TYK2/JAK1 inhibitor brepocitinib in patients with plaque psoriasis

*Journal of Pharmacokinetics and Pharmacodynamics*

Nikolaos Tsamandouras ^1^ • Ruolun Qiu ^1^ • Jim H. Hughes ^2^ • Kevin Sweeney ^2*^ • John P. Prybylski ^2^ • Christopher Banfield ^1*^ • Timothy Nicholas ^2^

^1^ Clinical Pharmacology, Early Clinical Development, Worldwide Research, Development and Medical, Pfizer, Cambridge, MA, USA
^2^ Clinical Pharmacology, Global Product Development, Pfizer, Groton, CT, USA
* At the time of the study

Corresponding author: Nikolaos Tsamandouras, PhD
Clinical Pharmacology, Early Clinical Development, Worldwide Research, Development and Medical, Pfizer,
1 Portland Street, Cambridge, MA, 02139, USA.
Tel: +1 857 225 2455. Email: [nikolaos.tsamandouras@pfizer.com](mailto:nikolaos.tsamandouras@pfizer.com)

# Online Resource 1

**NONMEM® Control Stream**

**$PROBLEM** run6.mod

**$INPUT** C STID=ID DOSE DAY DAYP PASI BPASI CL CAVE CFB TRTG=DROP AGE SEX RACE BWT PCFB PASI50 PASI75 PASI90 PASI100 TRTN CMT TIME PASITR1=DV PASITR2=DROP PASITR21=DROP PASITR22=DROP PASITR23=DROP

**$DATA** B7931004_PKPD_CAVE.csv

IGNORE=C

**$SUBROUTINES** ADVAN13 TOL=7

**$MODEL**

COMP = (PASI)

**$PK**

;----------- Define fixed effects ---------------

TVBASE = **THETA**(1) ; Baseline

TVIMAX = **THETA**(2) ; Imax (fixed to 1, included here just for completeness)

TVIC50 = **THETA**(3) ; IC50

TVKOUT = **THETA**(4) ; Kout

TVPMAX = **THETA**(5) ; Maximum placebo effect

TVKP = **THETA**(6) ; Rate of placebo effect onset

PHI = **THETA**(7) ; PHI is the precision parameter of the beta distribution

ZETA1 = **THETA**(8) ; ZETA1 is the intercept associated to the probability of 0 PASI

ZETA2 = **THETA**(9) ; ZETA2 is the slope associated to the probability of 0 PASI

;---------------- Add IIV ------------------------

LGBASE = **LOG**((TVBASE-12)/(72-TVBASE)) + **ETA**(1)

BASE = 12+(72-12)***EXP**(LGBASE)/(1+**EXP**(LGBASE))

IMAX = TVIMAX + **ETA**(2)

IC50 = TVIC50 * **EXP**(**ETA**(3))

KOUT = TVKOUT * **EXP**(**ETA**(4))

LGPMAX = **LOG**(TVPMAX/(1-TVPMAX)) + **ETA**(5)

PMAX = **EXP**(LGPMAX)/(1+**EXP**(LGPMAX))

KP = TVKP * **EXP**(**ETA**(6))

;----------- Indirect Response ODE ---------------

**A_0**(1) = 1

KIN = KOUT

**$DES**

**DADT**(1) = KIN*(1-(IMAX*CAVE/(IC50+CAVE))) -KOUT***A**(1)

;-------- Define Placebo and Drug effect ---------

**$ERROR**

PLEFF = BASE*PMAX*(1-**EXP**(-KP*TIME)) ; Placebo effect

DREFF = BASE*(1-PMAX)*(1-**A**(1)) ; Drug effect

NMUR = BASE - PLEFF - DREFF ; Combine placebo and drug effect

;----------- Use Beta distribution ---------------

MUR = NMUR / 72 ; Expected value of the beta (has to be between 0-1)

IPRED = MUR

ALPHA = MUR * PHI

BETA = (1-MUR)*PHI

;--- Nemes approximation to the gamma function ----

; Note: GAMLN function can be used instead in NONMEM 7.3 onwards)

X1 = ALPHA + BETA

X2 = ALPHA

X3 = BETA

LGAMMAX1 = X1*(**LOG**(X1)-1) + 0.5*(**LOG**(2*3.1415)-**LOG**(X1)) + (5/4)*X1***LOG**(1+(1/(15*X1**2)))

LGAMMAX2 = X2*(**LOG**(X2)-1) + 0.5*(**LOG**(2*3.1415)-**LOG**(X2)) + (5/4)*X2***LOG**(1+(1/(15*X2**2)))

LGAMMAX3 = X3*(**LOG**(X3)-1) + 0.5*(**LOG**(2*3.1415)-**LOG**(X3)) + (5/4)*X3***LOG**(1+(1/(15*X3**2)))

;---------- Probability for 0 PASI ------------

LGPZ = ZETA1 - ZETA2*MUR

PZ = **EXP**(LGPZ)/(1+**EXP**(LGPZ))

;---------- Log-likelihood ------------

**IF** (DV.EQ.0) **THEN**

LL = **LOG**(PZ)

**ELSE**

LL = LGAMMAX1 - LGAMMAX2 - LGAMMAX3 + (ALPHA-1)***LOG**(DV) + (BETA-1)***LOG**(1-DV) +**LOG**(1-PZ)

**ENDIF**

Y=-2*LL

;---- User-defined residuals for the beta ---------

SOR = (DV-IPRED)/**SQRT**(IPRED*(1-IPRED)/(1+PHI)) ;Standardized ordinary residuals

;---------------- Initial Estimates ---------------

**$THETA**

(0, 18, 72) ; BASE

1 FIX ; IMAX

(0, 40) ; IC50

(0, 0.06) ; KOUT

(0, 0.4, 1) ; PMAX

(0, 0.05) ; KP

(0, 40, 300) ; PHI

(2) ; ZETA1

(0,30) ; ZETA2

**$OMEGA**

1 ; BASE

0 FIX ; IMAX

0 FIX ; IC50

0 FIX ; KOUT

0 FIX ; PMAX

0 FIX ; KP

;-------------- EST, COV and TABLES ---------------

**$ESTIMATION** MAXEVAL=9999 PRINT=1 METHOD=COND -2LL NUMERICAL LAPLACIAN NOHABORT SIG=3 SIGL=12 FILE=run6.ext MSFO=msf_run6

**$COV** UNCONDITIONAL PRINT=E

**$TABLE** ID DOSE TRTN DAY DAYP PASI BPASI CAVE CFB AGE SEX RACE BWT PCFB PASI50 PASI75 PASI90 PASI100 PASITR1 MUR NMUR SOR BASE IMAX IC50 KOUT PMAX KP PHI ZETA1 ZETA2 ALPHA BETA Y LL PZ NOPRINT ONEHEADER NOTITLE FORMAT=,1PE16.8E3 FILE=sdtab_run6.csv

**$TABLE** ID BASE IMAX IC50 KOUT PMAX KP PHI **ETA**(1) **ETA**(2) **ETA**(3) **ETA**(4) **ETA**(5) **ETA**(6) BPASI AGE SEX RACE BWT NOPRINT FIRSTONLY NOAPPEND ONEHEADER NOTITLE FORMAT=,1PE16.8E3 FILE=patab_run6.csv

# Online Resource 2


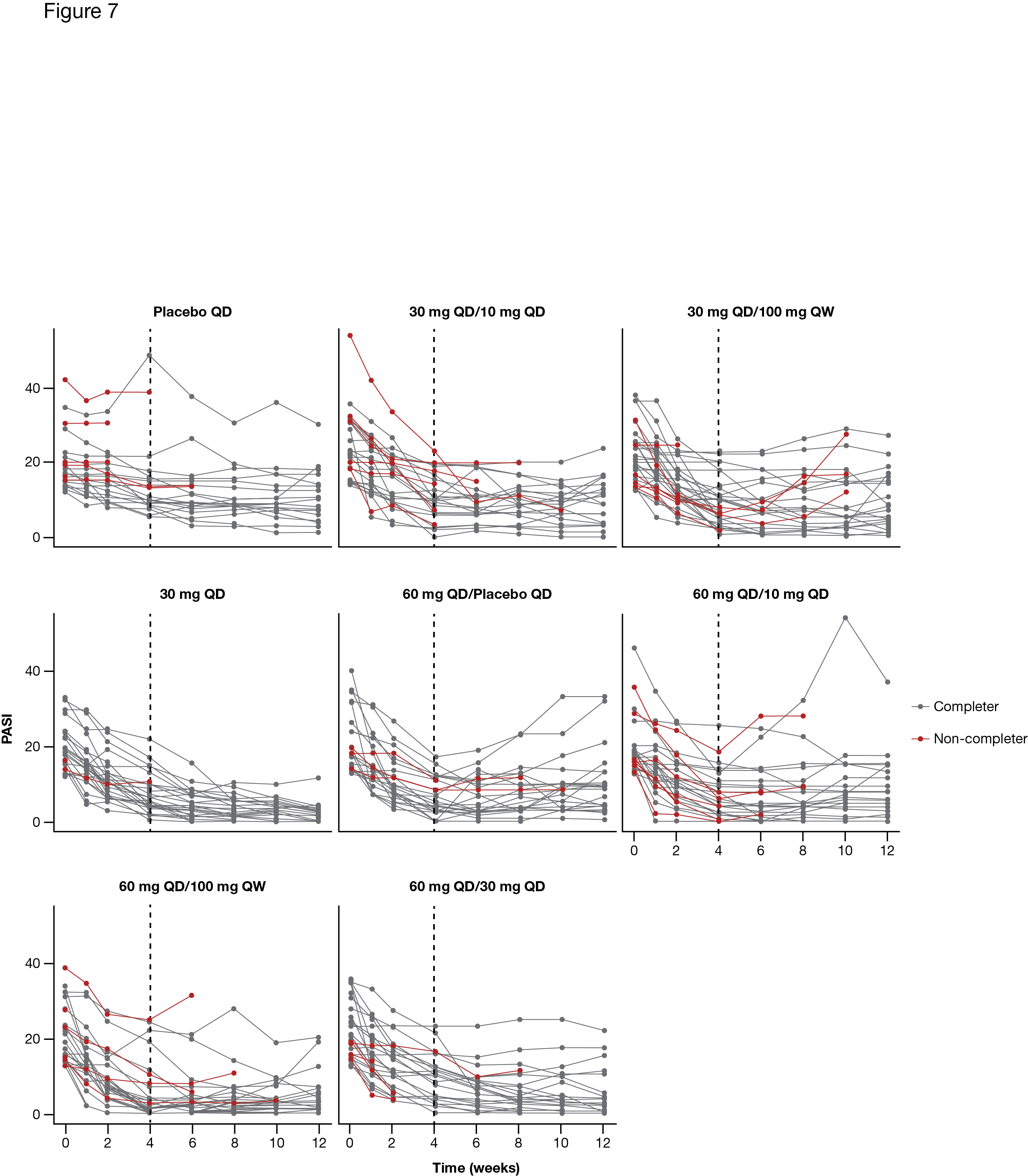
 **Fig S1.** Longitudinal individual PASI profiles stratified by treatment arm and color-coded by completer status. Grey line graphs depict participants that completed the 12-week treatment period, while red line graphs depict participants who dropped out before the end of the 12-week treatment period. The vertical dashed black line at 4 weeks indicates the transition from the induction to the maintenance treatment period. When a different dose was administered between the induction (up to week 4) and maintenance (weeks 5 through 12) periods, the respective regimens were reported, separated by “/”. PASI: Psoriasis Area and Severity Index, QD: once daily, QW: once weekly

# Online Resource 3


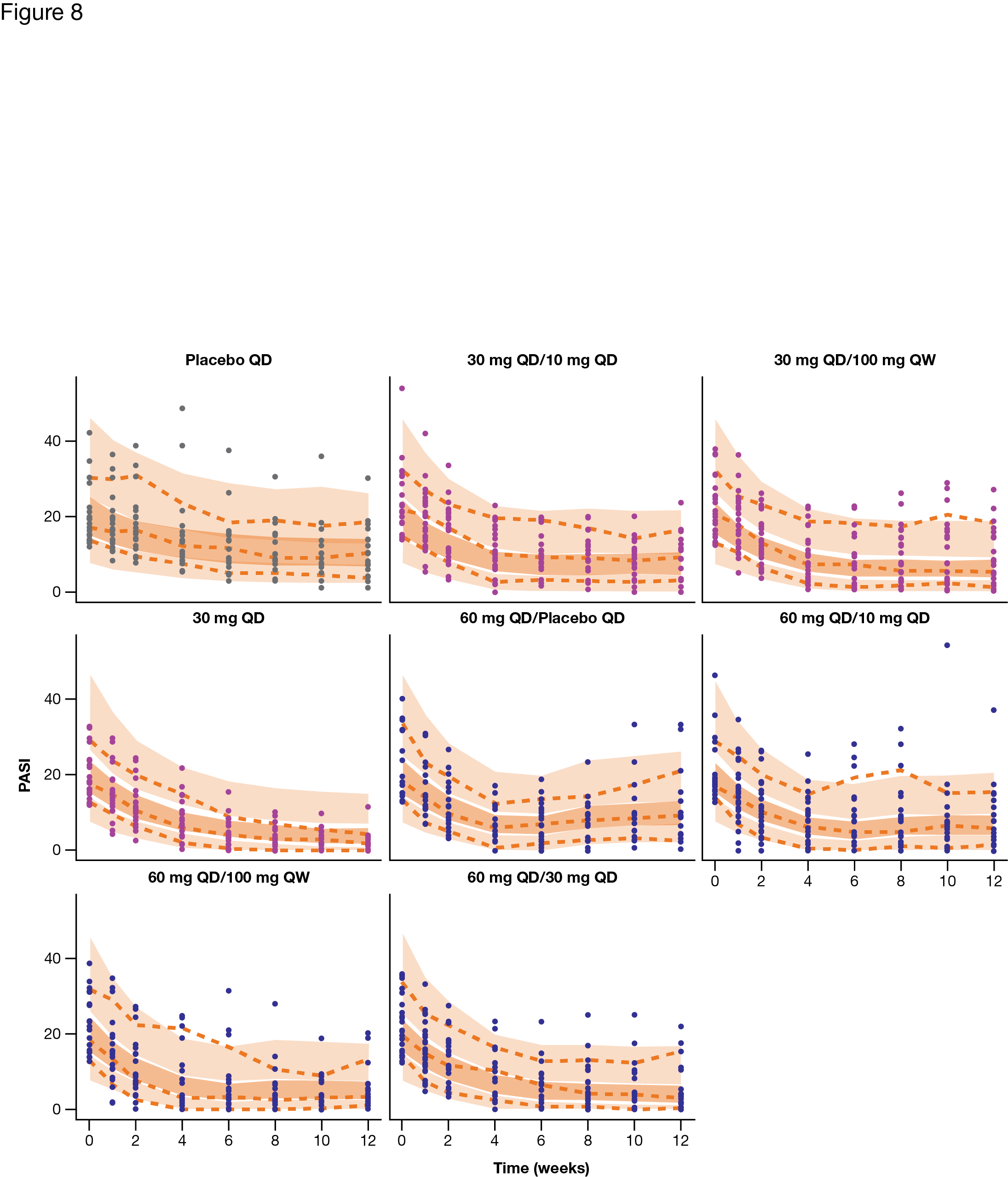


**Fig S2.** VPC stratified by treatment arm. Closed circles represent the observed individual PASI data. Orange dashed lines represent the 10^th^, 50^th^, and 90^th^ percentile of the observed data. Dark orange and light orange shaded areas represent 95% CIs around the model-derived median and model-derived 10^th^/90^th^ percentiles, respectively.
CI: confidence interval, PASI: Psoriasis Area and Severity Index, QD: once daily, QW: once weekly, VPC: visual predictive check

# Online Resource 4


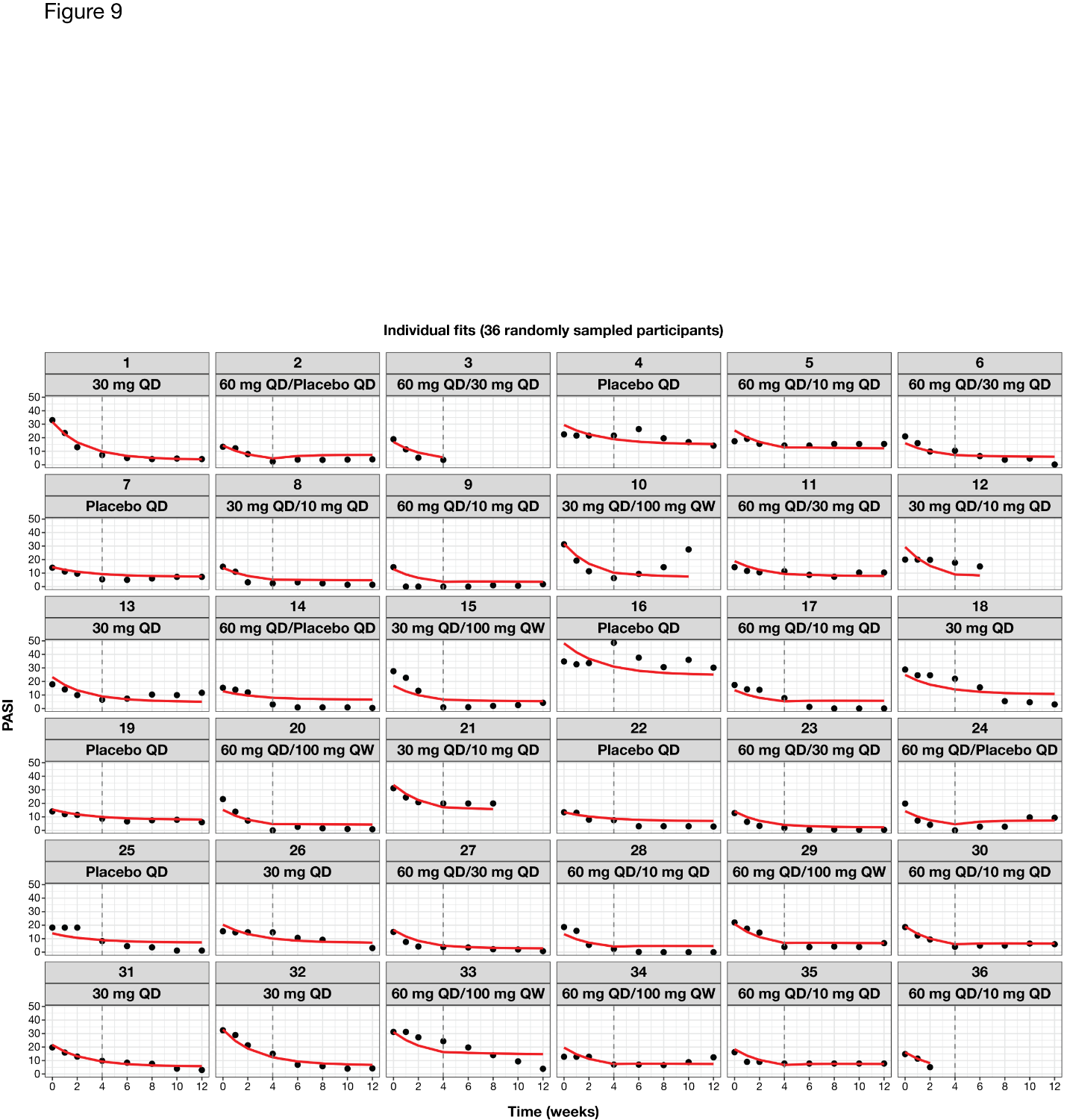

**Fig S3.** PASI individual model fits from 36 randomly sampled participants. Closed black circles represent observed data and red lines represent individual model predictions. The vertical dashed grey line at 4 weeks highlights the transition from the induction to the maintenance treatment period. When a different dose was administered between the induction (up to week 4) and maintenance (weeks 5 through 12) periods, the respective regimens were reported, separated by “/”. PASI: Psoriasis Area and Severity Index, QD: once daily, QW: once weekly
